# Heterozygous Deletion of α-Neurexin I or α-Neurexin II Results in Behaviors Relevant to Autism and Schizophrenia

**DOI:** 10.1037/bne0000108

**Published:** 2015-12

**Authors:** James Dachtler, Jose L. Ivorra, Tessa E. Rowland, Colin Lever, R. John Rodgers, Steven J. Clapcote

**Affiliations:** 1 School of Biomedical Sciences, University of Leeds; 2 Department of Psychology, Durham University; 3 Institute of Psychological Sciences, University of Leeds; 4 School of Biomedical Sciences, University of Leeds

**Keywords:** neurexin, Nrxn1, Nrxn2, social, autism

## Abstract

The neurexins are a family of presynaptic cell adhesion molecules. Human genetic studies have found heterozygous deletions affecting *NRXN1* and *NRXN2*, encoding α-neurexin I (Nrxn1α) and α-neurexin II (Nrxn2α), in individuals with autism spectrum disorders and schizophrenia. However, the link between α-neurexin deficiency and the manifestation of psychiatric disorders remain unclear. To assess whether the heterozygous loss of neurexins results in behaviors relevant to autism or schizophrenia, we used mice with heterozygous (HET) deletion of Nrxn1α or Nrxn2α. We found that in a test of social approach, Nrxn1α HET mice show no social memory for familiar versus novel conspecifics. In a passive avoidance test, female Nrxn1α HET mice cross to the conditioned chamber sooner than female wild-type and Nrxn2α HET mice. Nrxn2α HET mice also express a lack of long-term object discrimination, indicating a deficit in cognition. The observed Nrxn1α and Nrxn2α genotypic effects were specific, as neither HET deletion had effects on a wide range of other behavioral measures, including several measures of anxiety. Our findings demonstrate that the heterozygous loss of α-neurexin I and α-neurexin II in mice leads to phenotypes relevant to autism and schizophrenia.

Autism and schizophrenia are common cognitive disorders that have strong genetic components, with heritability estimates around 80% (Cardno & Gottesman, 2000; Ronald et al., 2006). Given the strong genetic component of both conditions, discovering which genetic loci are altered in autistic and schizophrenic patients represents an important part of understanding subsequent developmental and functional abnormalities within the brain.

One family of proteins that has gained recent prominence in both conditions is the neurexins, presynaptic cell adhesion molecules that are encoded by paralogous genes (*NRXN1-3*). Neurexins exist as either a longer α form, or a shorter β form, both of which are transcribed from independent promoters (Ichtchenko, Nguyen, & Sudhof, 1996). Neurexins play a role in synaptic function through their postsynaptic links to neuroligins and leucine-rich repeat transmembrane proteins (LRRTMs; Chubykin et al., 2007; Craig & Kang, 2007; de Wit et al., 2009; Missler et al., 2003). The first evidence linking neuropsychiatric disorders to heterozygous deletions affecting α-neurexin I (*NRXN1α*) was the discovery of a de novo deletion of the promoter and exons 1–5 of *NRXN1α* in a 7-year old boy with autism (Friedman et al., 2006), and a deletion of the promoter and exon 1 in two schizophrenic siblings, inherited from their unaffected mother (Kirov et al., 2009). Subsequently, heterozygous deletions affecting exons of *NRXN1* have been found in other cases of schizophrenia or autism (Reichelt, Rodgers, & Clapcote, 2012). Although currently unexplained, deletions within *NRXN1* have been associated with a variety of developmental disorders other than schizophrenia and autism, including mental retardation, language delay and hypotonia (Ching et al., 2010). More recently, heterozygous deletions affecting neurexin II (*NRXN2*) have been discovered in individuals with autism. A boy with autism and his father with severe language delay had a frameshift mutation within *NRXN2* exon 12 (Gauthier et al., 2011), whereas a 21-year-old man with a clinical phenotype including autistic traits, such as speech and language deficits and insistence on routine, had a 570-kb de novo deletion of 24 genes at chromosome 11q13.1, including *NRXN2* (Mohrmann, Gillessen-Kaesbach, Siebert, Caliebe, & Hellenbroich, 2011).

Previously, we used homozygous Nrxn2α knockout (KO) mice to assess the hypothesis that behavioral phenotypes related to autism could be associated with α-neurexin II deficiency. We found that Nrxn2α homozygous KO mice display a deficit in social responses—a core symptom of autism—as well as behaviors related to anxiety (Dachtler et al., 2014), which is often comorbid with autism (White, Oswald, Ollendick, & Scahill, 2009). Others have examined whether the loss of α-neurexin I in mice leads to phenotypes reflecting autism and schizophrenia. Relative to wild-type controls, Nrxn1α homozygous KO mice display heightened aggression toward, and less social investigation of, juvenile mice, while also expressing anxiety-like behavior in a light/dark box (Grayton, Missler, Collier, & Fernandes, 2013). These mice also have reduced prepulse inhibition (Etherton, Blaiss, Powell, & Sudhof, 2009), a measure of sensorimotor gating, which is also altered in schizophrenic and some autistic patients (Braff, Grillon, & Geyer, 1992; Perry, Minassian, Lopez, Maron, & Lincoln, 2007). Thus far, there have been few reports of patients with compound heterozygous mutations of *NRXN1*. A female suffering from Pitt-Hopkins-like syndrome-2, autistic traits, mental retardation, hyper-breathing and developmental regression inherited a deletion spanning exons 1–4 of *NRXN1* from her unaffected mother and a stop mutation in exon 15 from her father (Zweier et al., 2009).

Other studies have discovered compound heterozygous *NRXN1* deletions in patients with mental retardation, early developmental delay and epilepsy (Duong et al., 2012; Harrison et al., 2011; Utine et al., 2014). However, since deletions of *NRXN1* and *NRXN2* found in schizophrenia or autism are commonly heterozygous, it is important to explore whether mice with heterozygous knockout of either *Nrxn1* or *Nrxn2* display behavioral phenotypes associated with these disorders. Limited work using Nrxn1α heterozygous (HET) mice has been conducted thus far; one study reported a sex-dependent increase in responsiveness to novelty (Laarakker, Reinders, Bruining, Ophoff, & Kas, 2012), while another study found no effects of Nrxn1α HET deletion in tests of sociability, cognition or anxiety (Grayton et al., 2013). In the present study, we assessed behavioral performance of Nrxn1α HET and Nrxn2α HET mice in a range of behavioral tests for phenotypes relevant to the human disorders.

## Method

### Ethics

All procedures were approved by the University of Leeds Animal Ethical and Welfare Review Board and were performed under United Kingdom Home Office Project and Personal Licenses in accordance with the Animals (Scientific Procedures) Act, 1986.

### Animals

Full details of the animals, their background and housing can be found elsewhere (Dachtler et al., 2014). In brief, male B6;129-*Nrxn3*^*tm1Sud*^/*Nrxn1*^*tm1Sud*^/*Nrxn2*^*tm1Sud*^/J mice (JAX #006377) were purchased from the Jackson Laboratory as heterozygous KO at *Nrxn1*, homozygous KO at *Nrxn2* and wild-type at *Nrxn3*. These were outbred once to the C57BL/6NCrl strain (Charles River, Margate, United Kingdom) to obtain mice that were individually either Nrxn1α or Nrxn2α KO heterozygotes. Subsequently, HET knockout males were bred with wild-type females (cousin mating). The reason for this is that female wild-types without potentially deleterious social withdrawal phenotypes were expected to be more attentive mothers, which should “normalize” the early life social experiences of different wild-type and HET litters. This should potentially reduce the confounding effects of an abnormally behaving HET mother upon subsequent anxiety, social and cognitive testing of the offspring (Meaney, 2001). DNA extracted from ear biopsies was used for PCR-based genotyping according to the Jackson Laboratory Nrxn1 v5, Nrxn2 v5, and Nrxn3 v1 protocols (http://jaxmice.jax.org/strain/006377.html#genotype). The genotyping method for Nrxn2α can be found elsewhere (Dachtler et al., 2014). For Nrxn1α, the primers 5′-CTG ATG GTA CAG GGC AGT AGA GGA CCA-3′ (common forward), 5′-CGA GCC TCC CAA CAG CGG TGG CGG GA-3′ (WT reverse), and 5′-GAG CGC GCG CGG CGG AGT TGT TGA C-3′ (KO reverse) were used with HotShot Diamond (Clent Life Science, Stourbridge, United Kingdom) using the thermocycling program of: 94 °C for 5 min, followed by 35 cycles of 94 °C for 30 s, 70 °C for 60 s, and 72 °C for 60 s followed by 72 °C for 120 s. PCR products were visualized using agarose gel electrophoresis, with a 440-bp band indicating the wild-type (WT) allele, and a 390-bp band indicating the KO allele.

### Behavioral Testing

All behavioral experiments were conducted using young adults over 8 weeks of age. All subjects were handled several times a day for 1 week before testing. Balanced groups of male and female Nrxn1α and Nrxn2α HET mice and their WT littermates were tested in a battery of behavioral tests in the following order: open field, elevated plus-maze, forced swim, tail suspension, social interaction, emergence, novel object, prepulse inhibition, passive avoidance, and nest-building. For less stressful tests (e.g., open field), a minimum of 3 days intertest interval was provided, while 1–2 weeks was given after more stressful tests (e.g., passive avoidance). The estrus cycle was not measured for any of the female mice. However, it is likely that female mice when undertaking each test would have been at random stages of the estrus cycle, owing to the large sample sizes, multiple experiments, and housing of mice in individually ventilated cages. Unless otherwise stated, all experiments were recorded using Any-Maze tracking software (Stoelting, Wood Dale, IL). Not all mice underwent the full series of experiments; a minority was not included in the final three experiments as they were required for breeding for other studies that would not have been possible had the mice undertaken the full battery of tests. For all experiments, 24 WT (m = 10, f = 14), 16 Nrxn1α HET (m = 6, f = 10), and 28 Nrxn2α HET (m = 15, f = 13) mice were used. For the emergence test, novel object, prepulse inhibition (PPI), and passive avoidance tests, 24 WT (m = 10, f = 14), 16 Nrxn1α HET (m = 6, f = 10), and 22 Nrxn2α HET (m = 13, f = 9) mice were used. For the WT group, a 50:50 ratio of mice were tested from the Nrxn1α HET and Nrxn2α HET breeding. Power calculation for two-way analysis of variance (ANOVA; sex vs. genotype), assuming η^2^ = 0.14, α = .05, and 1-β = 0.95, revealed that a sample size of 20 per sex per genotype is required. Although we were unable to test these numbers, our groups are nevertheless suitably powered to detect genotypic differences (Crawley, 2008).

Experiments were conducted using identical protocols and apparatus to those in (Dachtler et al., 2014), where full methodological details can be found. For all experiments, except prepulse inhibition and passive avoidance (that were illuminated by LEDs within the chambers), the apparatus was illuminated by standard fluorescent ceiling lights at an intensity of ∼200 Lux. Methods are briefly described below.

### Open Field

Mice were placed inside a 40 × 40 × 40 cm arena, and allowed to explore for 30 min. Tracking software divided the arena floor into three zones: outer (8 cm from the walls), center (6.4 cm^2^) and intermediate (the remaining area). Time spent in, and entries made to, each of the zones and distance traveled were measured. Rearing and grooming events were recorded during the trial by the experimenter. Lighting of the maze was provided by standard fluorescent ceiling lights, with intensity measured using a lux meter at ∼200 Lux at the center of the arena. A tripod supporting a webcam was positioned directly above the arena to record the trials.

### Elevated Plus Maze

The maze consisted of two closed arms that had white opaque walls and two open arms, connected by a central zone. All arms measured 30 cm long and 5 cm wide. The mouse was placed into the central zone and allowed to explore for 5 min. The number of entries and time spent in all the zones was measured. Head dips from the central zone and open arms were scored manually online by the experimenter.

### Forced Swim Test

A 5 liter glass beaker was filled with 3 liters of water at 25 ± 1 °C. The mouse was placed into the water and left for 6 min. The time spent floating was measured during the final 4 min of the trial. Floating was defined by the absence of movements except those required to keep the mouse afloat.

### Tail Suspension Test

The mouse was suspended by the tail using electrical tape affixed to a structure 20 cm above a table. During a 6-min trial, immobility was measured, classified as the complete absence of movement except for respiration.

### Three Chamber Social Approach Test

Social preference was measured using a three-chambered arena. Following a 15-min habituation period, two cages (10 cm W × 10.5 cm H, consisting of vertical metal bars separated by 9 mm) were placed into the left and right chambers of the arena. One contained an unfamiliar male C57BL/6 mouse (age >10 weeks; “Stranger 1”) whereas the other remained empty. Time spent exploring either cage was measured for 10 min. This is considered a test of “sociability” (Moy et al., 2004; Yang, Silverman, & Crawley, 2011). Thereafter, the previously empty cage had a second unfamiliar male mouse placed into it (age >10 weeks; “Stranger 2”) and the time exploring either Stranger 1 or Stranger 2 was measured for 10 min. This is considered a test of “social approach”’ (Yang et al., 2011) and likely involves both cognitive and motivational components.

### Emergence Test

A black enclosure (17 cm L × 11 cm W × 5.5 cm H) was placed against the middle of a wall within an open field arena (40 × 40 × 40 cm). The enclosure had a 6 × 3.5 cm opening to allow the mouse free access between the enclosure and the arena. The experiment started by placing the mouse within the enclosure. Latency to emerge from the enclosure and the total time spent outside the enclosure were measured across the 15 min trial.

### Novel Object Test

Mice were first habituated to an open field arena (as used for the open field test). On Days 1, 2, and 3, each mouse was placed into the empty arena for 15 min and allowed to explore freely. On Day 4, mice were given a 10 min habituation trial, after which they returned to the arena, which now contained two novel, but identical plastic objects (Mega Bloks, 6 × 6 × 4 cm (total) with four 2.5 × 2 cm cylinders on the top) placed 7 cm away from each opposing corner of one wall of the arena. The placement of the objects was varied during the experiment. Mice were allowed to explore the objects for 10 min, after which the mice were returned to their home cage. Twenty-four hours later, the mouse was returned to the arena containing one of the previously explored objects and a novel object. Discrimination was assessed by comparing the total contact time of the mouse with each object. Exploration was defined as either direct contact (nose poke or touch) or exploration within close proximity (2 cm); accidental touches or behaviors such as sitting near the object were not counted.

### Prepulse Inhibition

Mice were tested for acoustic startle reactivity (ASR) and PPI using the SR-LAB startle response system (San Diego Instruments, San Diego, CA). Acoustic startle responses were measured at 70, 80, 85, 90, and 120 dB. PPI was measured by the delivery of a tone at either 70, 80, 85, and 90 (prepulse) for 10 ms followed by a 100 ms gap at background noise and then a 120 dB “startle” tone for 40 ms. Background noise at 65 dB was presented throughout the trials. Intratrial intervals were averaged at 25 s.

### Passive Avoidance

Passive avoidance was assessed using a Med Associates Shuttle Box under the control of Med PC software. One half of the chamber was darkened using a black cloth cover (defined as the “dark chamber”), whereas the other side was illuminated by a white light. During the training phase, latency to cross from the light chamber to the dark chamber was measured (Baarendse et al., 2008). Once crossed into the dark chamber, the mouse received a 3-s 0.45-mA footshock. Twenty-four hours later, the mouse was placed back into the light chamber and the latency to cross to the dark chamber measured. The trial was stopped either if the mouse had not crossed within 300 s or when the mouse had traversed to the dark chamber.

### Nesting Behavior

Details of the methodology have been previously described (Deacon, 2006). Mice were individually housed before the dark cycle with no enrichment except for a 3 g “nestlet” (Lillico, Surrey, United Kingdom) of pressed cotton (other nestlets were cut to make the weight up to 3 g, when necessary). Nesting was assessed the following morning at 0800 hours and was rated using the following scale:

1 Nestlet not noticeably touched (>90% intact).
2 Nestlet partially torn up (50–90% intact).
3 Nestlet mostly shredded but no identifiable nest site.
4 Identifiable nest, but flat.
5 A perfect or near-perfect nest.

For this test, 19 WT (m = 8, f = 11), 12 Nrxn1α HET (m = 4, f = 8), and 16 Nrxn2α HET (m = 10, f = 6) mice were used. Nests were photographed within the cage and subsequently scored by a second experimenter. No significant differences were observed between nesting scores (Wilcoxon signed-ranks test, *p* > .05, data not shown), suggesting that while this is a subjective approach to quantitatively scoring of nesting ability, it is a robust, replicable measurement.

### Western Blotting

WT (*n* = 4, m = 2, f = 2 [1 male and 1 female from Nrxn1 breeding and 1 male and 1 female from Nrxn2 breeding]), Nrxn1α HET (*n* = 4, m = 2, f = 2), and Nrxn2α HET (*n* = 4, m = 2, f = 2) mice were killed by CO_2_ asphyxiation and their brains were quickly extracted and snap frozen in liquid N_2_ and stored at −80 °C. The hippocampus from both hemispheres was dissected under microscope on ice. The tissue was homogenized, the supernatant aliquoted, and protein concentration measured by Bradford assay. Samples were stored at −80 °C. Aliquots of 30 μg total protein were prepared for loading by the addition of Laemmli sample buffer (Bio-Rad, Hemel Hempstead, United Kingdom) with 5% β-mercaptoethanol, and incubated at 95 °C for 5 min.

Samples were subjected to gradient sodium dodecyl sulfate-polyacrlyamide gel electrophoresis (SDS-PAGE) on polyacrylamide gels (4–15%; Mini-PROTEAN TGX, Bio-Rad, Hemel Hempstead, United Kingdom), transferred to polyvinylidene fluoride (PVDF) membranes (Pall, Portsmouth, United Kingdom), and blocked for either 1 hr at room temperature (reaction time, RT) or overnight at 4 °C in 5% milk in 1 × phosphate-buffered saline (PBS) with 0.05% Tween-20. Membranes were incubated with anti-Munc18-1 (sc-14557; Santa Cruz, Heidelberg, Germany) at the concentration of 1:1000 in 5% milk for 1.5 hr at RT. Antigoat (sc-2020; Santa Cruz) horseradish peroxidase (HRP)-linked secondary antibodies were incubated in 5% milk for 1 hr at RT. Bound peroxidase-conjugates were visualized using enhanced chemiluminescence (ECL) Western Blotting Substrate (Promega, Southampton, United Kingdom). To confirm equal loading, membranes were immersed in stripping buffer at 50 °C for 30 min before incubating with 1:5000 anti-β-actin (A1978; Sigma, Poole, United Kingdom) and the antimouse (sc-2371; Santa Cruz) secondary. All western blots were repeated a minimum of three times. Densitometry was performed using ImageJ (v1.46; http://imagej.nih.gov/ij), with expression normalized to the β-actin loading control.

### Data Analysis

All data are expressed as mean ± *SEM*. Data were tested for normality and homogeneity of variance using SPSS (Chicago, IL). If these conditions were not met, then the Geisser-Greenhouse correction was used. To assess differences between the variables and their impact upon performance, two-sample *t* tests or ANOVAs were conducted. Performance across time bins was analyzed by repeated measures (RM) ANOVA. If there were significant interactions between variables, tests of simple main effects were performed (with the use of Bonferroni correction for multiple comparisons), followed by post hoc analysis where necessary. To test for differences in nest building rating, the Kruskal-Wallis ANOVA was used. All analyses and graphs were made using SPSS version 20 or GraphPad Prism version 6. In all cases, α was set at ≤0.05. Sex effects were tested for all experiments; however, only one significant result was found (in the passive avoidance test). Main effects of sex and interactions with sex for all other experiments were *p* > .05, and therefore, both sexes were combined into genotypic groups.

## Results

### α-Neurexin HET Mice Have Altered Social Behaviors

**Figure 1 fig1:**
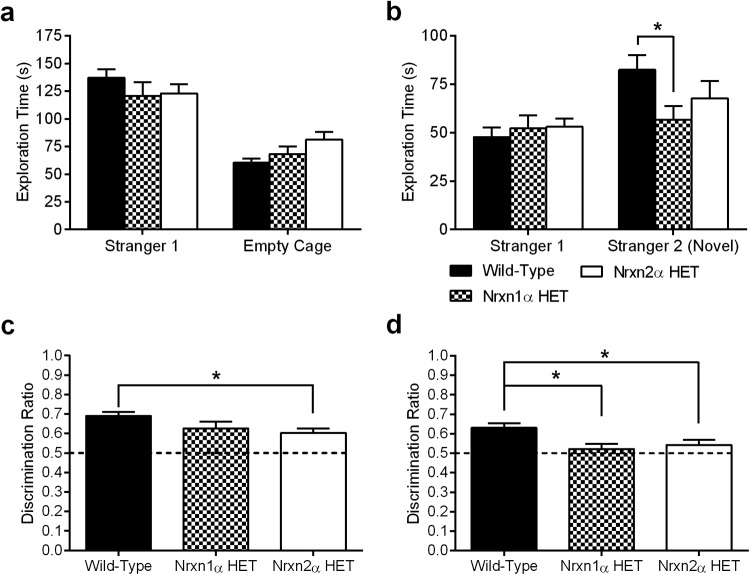
Impaired social behavior in Nrxn HET mice. When given the choice between exploring a cage containing a novel conspecific versus an identical empty cage (a), wild type (WT; *n* = 24), Nrxn1α HET (*n* = 16), and Nrxn2α HET (*n* = 28) mice spent a similar amount of time exploring both the novel mouse and the empty cage. In the second stage (b), Nrxn1α HET mice were unable to discriminate between the previously explored mouse (“Stranger 1”) and a second novel conspecific (“Stranger 2”). Compared with WT, Nrxn1α HET mice spent significantly less time exploring Stranger 2. When expressed as a discrimination ratio, Nrxn2α HET mice have significantly weaker discrimination between Stranger 1 and the empty cage (c). Discrimination between Stranger 1 and Stranger 2 was significantly weaker for both Nrxn1α HET and Nrxn2α HET mice compared with WT (d). HET = heterozygous; Nrxn1α = α-neurexin I; Nrxn2α = α-neurexin II. * *p* < .05.

In light of the altered social behavior exhibited by patients with autism, and the altered social investigative behavior of both Nrxn1α and Nrxn2α homozygous KO mice (Dachtler et al., 2014; Grayton et al., 2013), we assessed willingness of Nrxn1α and Nrxn2α HET mice to socially approach novel conspecifics in a three-chambered assay for sociability. This test gives the mouse the choice of whether to spend time exploring the chamber containing an unfamiliar mouse within a wire cage (Stranger 1) or the opposite chamber containing an identical but empty cage (Moy et al., 2004). Both Nrxn1α and Nrxn2α HET mice showed a statistically indistinguishable amount of time exploring both the cage containing the novel conspecific and the empty cage compared with WT mice (Figure 1a; RM ANOVA, genotype [*F*(2, 62) <1), Genotype × Discrimination (*F*(2, 62) = 2.93, *p* = .069]).[Fig-anchor fig1]

Directly after the initial test of sociability, the mice were subjected to a second novel conspecific; preference for exploring the new unfamiliar mouse (Stranger 2) versus the original unfamiliar mouse (Stranger 1) was measured (Figure 1b). A preference for exploring Stranger 2 was shown by WT and Nrxn2α HET mice, but not by Nrxn1α HET mice, which spent significantly less time than WT mice exploring Stranger 2 (RM ANOVA, significant Genotype × Discrimination interaction (*F*(2, 62) = 20.13, *p* < .0001). Compared with WT mice, pairwise comparisons confirmed that Nrxn1α HET mice spent significantly less time exploring Stranger 2 (*p* = .044). There were no significant differences for exploration of the cage containing the previously explored mouse (*p* = .91). When analyzed for within-genotype differences between the Stranger mice, only wild-types showed a significant difference for exploring Stranger 2 compared with Stranger 1 (*p* = .0001; Nrxn1α HET mice *p* = .96, Nrxn2α HET mice *p* = .15). General ambulation was similar across the genotypes for all three stages of the test (Fig. S1a–c). Previously, we and others have shown that both Nrxn1α and Nrxn2α KO mice have intact olfaction (Dachtler et al., 2014; Grayton et al., 2013), suggesting that any altered social exploration is not because of a reduced ability to detect social odors.

While direct contact time with the cages revealed only subtle differences between the genotypes, both Nrxn1α HET and Nrxn2α HET mice appear to have weaker social discrimination during the two stages. When expressed as a discrimination ratio between time spent exploring the two cages, significant differences were revealed between the genotypes. During the social approach phase, Nrxn2α HET mice have significantly weaker discrimination between Stranger 1 and the empty cage (Figure 1c; one-way ANOVA *F*(2, 62) = 3.82, *p* = .027, pairwise comparison: WT vs. Nrxn2α HET *p* = .023). During the social preference test, both Nrxn1α and Nrxn2α HET mice have weaker discrimination between Stranger 1 and Stranger 2 (Figure 1d; one-way ANOVA *F*(2, 62) = 4.83, *p* = .011, pairwise comparison: WT vs. Nrxn1α Het *p* = .021, WT vs. Nrxn2α HET *p* = .033).

Previously it has been shown that Nrxn1α KO mice have a reduced ability to make nests within their home cage (Etherton et al., 2009; Grayton et al., 2013), a phenotype that has been linked to reduced maternal care and hence a measure of social behavior (Peripato & Cheverud, 2002), and that females show maternal indifference to their own pups (Geppert et al., 1998). We found that Nrxn1α and Nrxn2α HET mice were able to make cotton nests of a similar quality to WT mice (Supplemental Material Figure 1d; Kruskal-Wallis ANOVA = 1.57, *p* = .46).

### α-Neurexin HET Mice Show Limited Impairments in Cognition

Schizophrenia patients frequently have deficits in their ability to form spatial memories (Piskulic, Olver, Norman, & Maruff, 2007) and, more generally, have lower IQ scores than healthy controls (Aylward, Walker, & Bettes, 1984; Hedman, van Haren, van Baal, Kahn, & Hulshoff Pol, 2013). Similarly reduced IQ scores have been noted in patients with autism, with up to 40% having an IQ low enough to be classified as severe to profound intellectual disability (S/PID; Fombonne, 2003). Patients suffering from mental retardation without autism or schizophrenia have also been found to harbor *NRXN1* deletions (Ching et al., 2010).

**Figure 2 fig2:**
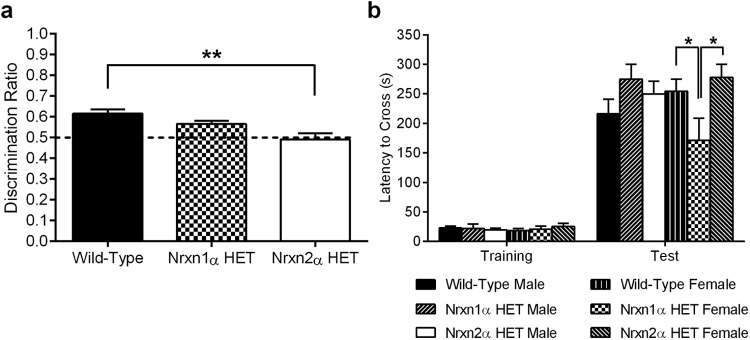
Nrxn2α HET mice lack object discrimination memory. In a test where mice had to discriminate between a previously explored object and a novel object following a 24 hour delay (a), Nrxn2α HET (*n* = 22) mice spend approximately the same amount of time exploring both objects, while wild type (WT, *n* = 24) and Nrxn1α HET (*n* = 16) mice display preference for the novel object. In another test of cognition, the passive avoidance test (b), all genotypes were able to correctly recall the aversive memory of crossing to the dark chamber where they previously received a footshock and inhibit their crossing latency, except for female Nrxn1α HET mice which crossed to the dark chamber significantly sooner than female Nrxn2α HET mice and female WT mice. HET = heterozygous; Nrxn1α = α-neurexin I; Nrxn2α = α-neurexin II. * *p* < .05. ** *p* = 0.0007.

To assess whether cognition was altered by the loss of α-neurexin, we tested Nrxn1α and Nrxn2α HET mice in two tests of long-term memory. Novel object discrimination requires the subject to discriminate between a previously explored object and a novel object. Nrxn1α KO mice were previously shown to be able to discriminate between familiar and novel objects after a 24-hr delay over the first half of a 10 min test, although discrimination was at chance for the second half of the test (Laarakker et al., 2012). Therefore, we tested Nrxn1α and Nrxn2α HET mice for the ability to discriminate novel objects after 24 hr. We found that Nrxn1α HET and WT mice spent a comparable amount of time exploring the novel object. However, Nrxn2α HET mice showed no preference for exploring the novel object (Figure 2a; one-way ANOVA, *F*(2, 62) = 7.74, *p* = .001, pairwise comparison reveals a significant difference between WT and Nrxn2α HET mice [*p* = .0007] but not between WT and Nrxn1α HET mice [*p* = .34]), despite all genotypes spending similar amounts of time exploring the objects during the training and test phases (Supplemental Material Figure 2a–b). When the contact time for the familiar and novel object was examined separately, Nrxn2α HET mice still showed no object discrimination (Supplemental Material Figure 2c).[Fig-anchor fig2]

Previously, we showed that Nrxn2α KO mice have no impairments within the passive avoidance test (Dachtler et al., 2014), and, consistent with that finding, Nrxn2α HET mice in the current study had similar retention latencies to WT mice (Figure 2b). However, female Nrxn1α HET mice transverse to the chamber where they received the footshock significantly sooner than female WT and Nrxn2α HET mice (RM ANOVA, significant Genotype × Sex × Latency interaction (*F*(2, 56) = 4.06, *p* = .023). Tests of simple main effects found a significant effect of genotype, sex, and latency for females in the test (*F*(2, 56) = 4.99, *p* = .01) but not conditioning phase (*F*(2, 56) <1) or for males (conditioning phase: *F*(2, 56) <1, test phase: *F*(2, 56) <1). Pairwise comparisons for the test phase: female WT versus female Nrxn1α HET *p* = .045, female Nrxn1α HET versus Nrxn2α HET *p* = .03. Together, female Nrxn1α HET mice exhibit an impairment in cognition when using aversive stimuli.

### α-Neurexin HET Mice Have Unaltered Ambulation and Anxiety

It has been noted that a subset of autistic patients have comorbid anxiety disorders (White et al., 2009), and a strong anxiety-like phenotype has been observed in Nrxn2α KO mice (Dachtler et al., 2014) and to a lesser extent in Nrxn1α KO mice (Grayton et al., 2013). Thus, we measured anxiety in the HET mice in three tests of exploratory behavior.

**Figure 3 fig3:**
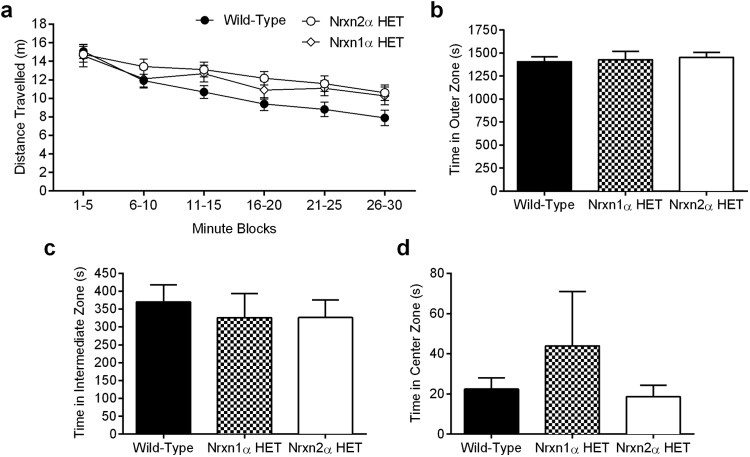
Nrxn HET mice exhibit no alterations in exploratory behavior within a novel environment. Over the 30 min of the test, general ambulation was similar between wild type (WT; *n* = 24), Nrxn1α HET (*n* = 16), and Nrxn2α HET (*n* = 28) mice (a). During the experiment, mice were automatically tracked and their propensity for spending time in different zones was measured. All three genotypes spent a similar amount of time in the outer zone (b), the intermediate zone (c) and the center zone (d). HET = heterozygous; Nrxn1α = α-neurexin I; Nrxn2α = α-neurexin II.

The open field test measures the propensity of a mouse to explore the relative safety of the arena walls versus the brightly lit center, a behavior called thigmotaxis. Increased thigmotaxis is thought to reflect increased anxiety in mice. Over the 30-min trial, general ambulation within the arena was similar between the genotypes (Figure 3a; RM ANOVA; genotype [*F*(2, 62) = 2.17, *p* = .12], time block [*F*(5, 310) = 29.37, *p* < .0001], interaction between the factors [*F*(10, 310) = 1.28, *p* = .24]), which included the first 5-min block, whereas others have shown greater distances traveled by Nrxn1α HET mice (Laarakker et al., 2012). Nrxn1α HET, Nrxn2α HET, and WT mice also spent a similar proportion of time in the outer (one-way ANOVA, *F*(2, 62) <1), intermediate (one-way ANOVA, *F*(2, 62) <1), and center zones (one-way ANOVA, *F*(2, 62) = 1.09, *p* = .34; Figure 3b–d), with no changes in the number of entries made into each zone (Supplemental Material Figure 3a–c; see legend for statistics). Grooming and rearing behavior was also not altered in either HET genotype (Supplemental Material Figure 3d–e; see legend for statistics).[Fig-anchor fig3]

**Figure 4 fig4:**
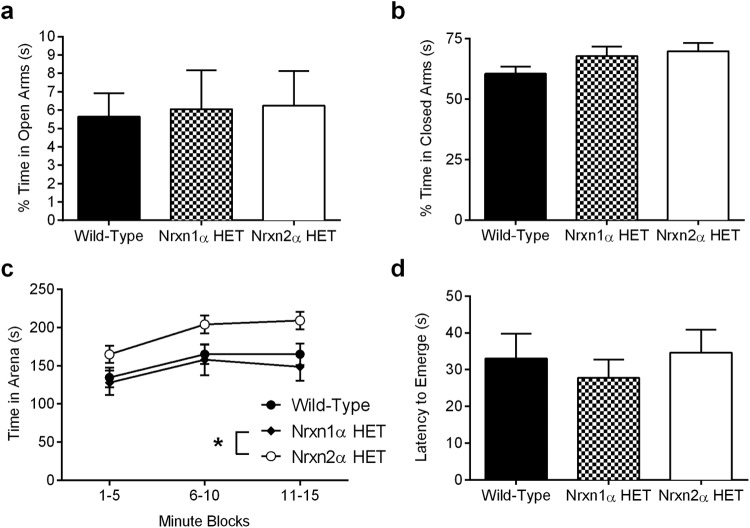
Nrxn HET mice do not display anxiety-like behaviors. Anxiety was assessed within the elevated plus maze by the propensity of mice to explore open arms. Wild type (WT; *n* = 24), Nrxn1α HET (*n* = 16), and Nrxn2α HET (*n* = 28) mice spent a statistically similar amount of time in the open arms (a) and the closed arms (b). In the emergence test, all genotypes spent similar proportions of their time out in the arena compared with WT mice. However, Nrxn2α HET (*n* = 22) mice spent a significantly longer proportion of their time out in the arena as compared with Nrxn1α HET mice but not WT (c). However, the latency to emerge initially from the enclosure was similar between the genotypes (d). HET = heterozygous; Nrxn1α = α-neurexin I; Nrxn2α = α-neurexin II. * *p* < .05.

The elevated plus maze tests the preference of mice to explore the relative safety of the enclosed arms of a novel environment versus brightly lit open arms. Mice tend to avoid the open arms, but exploration of the open arms can be bidirectionally increased or decreased with anxiolytic or anxiogenic drugs (Lister, 1987; Pellow & File, 1986). Both Nrxn1α and Nrxn2α HET mice spent similar amounts of time exploring the open arms and the closed arms (one-way ANOVA, *F*(2, 62) <1 and *F*(2, 62) = 2.37, *p* = .11, respectively; Figure 4a–b). Nrxn2α HET mice spent significantly less time in the center of the plus maze compared with WT mice (one-way ANOVA, *F*(2, 62) = 4.54, *p* = .014, pairwise comparisons: WT vs. Nrxn2α HET *p* = .013; Supplemental Material Figure 4a); however, the genotypes did not differ on any other measure recorded, including distance traveled, exploratory head dips, and entries made into the arms (Supplemental Material Figure 4a–f; see legend for statistics).[Fig-anchor fig4]

Finally, we examined anxiety-like behaviors in the emergence test, in which we previously showed that Nrxn2α KO mice have a preference for remaining within the enclosure instead of exploring a brightly lit open arena (Dachtler et al., 2014). All genotypes had similar latencies to emerge from the enclosure (one-way ANOVA, *F*(2, 56) <1). Both neurexin groups spent similar amounts of time in the open arena, compared with WT mice (though Nrxn2α HET arena time was significantly more than that of the Nrxn1α HET mice) (RM ANOVA; genotype [*F*(2, 56) = 3.45, *p* = .039], time block [*F*(2, 112) = 14.11, *p* < .0001], interaction between the factors [*F*(4, 112) <1]. Pairwise comparison: WT versus Nrxn1α HET mice: *p* = .84, WT versus Nrxn2α HET mice: *p* = .066, Nrxn1α HET versus Nrxn2α HET mice: *p* = .037 (Figure 4c–d).

Given that depression-related behaviors have been reported in other mouse models of schizophrenia (Clapcote et al., 2007; Hikida et al., 2007), we tested Nrxn1α and Nrxn2α HET mice in the Porsolt forced swim test and tail suspension test, but found no significant differences compared with WT mice (Supplemental Figure 5a–b; see legend for statistics).

### α-Neurexin HET Mice Show Intact Prepulse Inhibition

**Figure 5 fig5:**
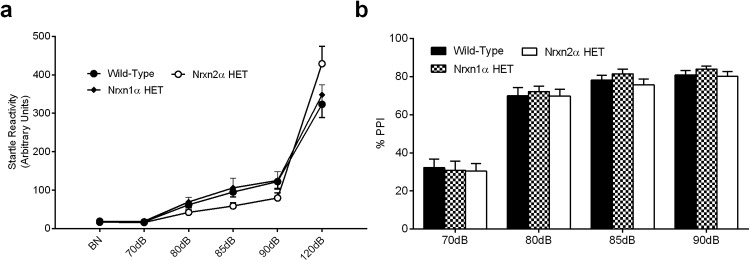
Prepulse inhibition is normal in Nrxn HET mice. Acoustic startle reactivity was measured at a variety of intensities up to 120 dB from background noise (BN) (a). Wild type (WT; *n* = 24), Nrxn1α HET (*n* = 16), and Nrxn2α HET (*n* = 23) mice displayed similar startle responses at all decibels. No significant differences were observed between the genotypes for the percentage of prepulse inhibition (PPI) inhibition across the different prepulse tones (b). HET = heterozygous; Nrxn1α = α-neurexin I; Nrxn2α = α-neurexin II.

Prepulse inhibition (PPI) is the ability to inhibit a startle response to a loud acoustic stimulus by receiving a quieter tone directly preceding it, and is thought to reflect sensorimotor gating. This ability has been found to be reduced in schizophrenic and some autistic patients (Braff et al., 1992; Perry et al., 2007), where the startle response remains unaltered by the preceding tone. This deficit has also been reported in Nrxn1α KO mice (Etherton et al., 2009). Nrxn2α Het mice had a marginally stronger startle response to the 120-dB startle tone, although no significant differences were observed at any of the sound intensities (RM ANOVA; significant Genotype × dB intensity interaction (*F*(10, 320) = 2.67, *p* = .022); pairwise comparison failed to reveal significant differences at any sound intensity between the genotypes (all *p* > .05; Figure 5a). No significant differences in PPI were found between the genotypes (RM ANOVA; genotype (*F*(2, 57) <1), dB (*F*(3, 201) = 309.9, *p* < .0001), and interaction between the factors (*F*(10, 171) <1; Figure 5b).[Fig-anchor fig5]

### Munc18-1 Expression Is Not Altered in α-Neurexin HET Mice

**Figure 6 fig6:**
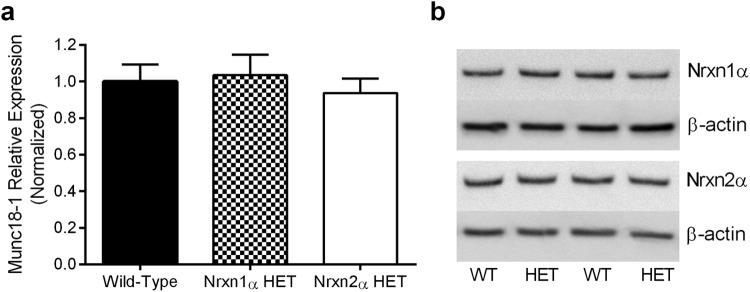
Hippocampal Munc18-1 expression is not altered Nrxn HET mice. Within the hippocampus, Munc18-1 expression was probed by western blotting (a and b). Compared with wild type (WT) mice (*n* = 4), Nrxn1α HET (*n* = 4), and Nrxn2α HET (*n* = 4) mice had statistically similar protein abundance of Munc18-1.

In addition to binding with postsynaptic neuroligins and LRRTMs, neurexins interact with various presynaptic proteins, of which one example is Munc18-1 (Biederer & Sudhof, 2000). The deletion of neuroligin-1 resulted in the reduced abundance of Munc18-1 in whole brain extracts from in neuroligin-1 KO mice (Blundell et al., 2010). In Nrxn2α KO mice, we observed significantly reduced expression in the hippocampus but not the cortex (Dachtler et al., 2014), suggesting a potential disruption of neurotransmission release (Verhage et al., 2000). To determine whether the protein abundance of Munc18-1 was altered in Nrxn1α and Nrxn2α HET mice, we tested homogenates of hippocampal tissue by western blotting. Compared with WT mice, we observed no significant difference in hippocampal Munc18-1 expression for either genotype (ANOVA; genotype (*F*(2, 33) <1; Figure 6a–b).[Fig-anchor fig6]

## Discussion

Deletions within the neurexin genes represent a contributory factor in both autism and schizophrenia. However, understanding how these genetic deletions result in behavioral traits associated with each disorder is lacking. In the current study, we used mice with heterozygous deletions of either Nrxn1α or Nrxn2α to determine whether behaviors relevant to both disorders would be affected in these mice. We found that in tests of sociability, Nrxn1α HET mice had deficits in the social preference task. Nrxn2α HET mice also showed no long-term (24 hr) discrimination for novel objects, but were unimpaired in the passive avoidance test. However, female Nrxn1α HET mice were significantly impaired in the passive avoidance test compared with female WT and Nrxn2α HET mice. The lack of impairments in other behavioral tests, including PPI, open field and emergence, points to specific effects that the deletion of either gene is likely to have in the genesis of behaviors relevant to schizophrenia and autism.

### Relationship to Other Studies of α-Neurexin Mice

Given that neurexin alterations were first discovered in autism and schizophrenia in 2006 (Friedman et al., 2006) and 2009 (Kirov et al., 2009), respectively, and further associations have been found since then (Reichelt et al., 2012), limited research has been conducted to determine whether neurexin deletions result in behaviors associated with these disorders. Within the social behavioral domain, which is most relevant to the symptomatology of autism, the first study to examine behaviors in Nrxn1α KO mice found impaired nest building ability (Etherton et al., 2009), which was interpreted as relevant to social and/or parental behaviors. The only other study thus far to examine Nrxn1α homozygous KO mice found increased sociability and social recognition to a novel versus familiar mouse in the three-chambered social approach test, although Nrxn1α KO mice displayed more aggression toward a juvenile conspecific (Grayton et al., 2013). That study also examined Nrxn1α HET mice and, in tests of sociability and social approach, found no significant differences from WT mice, including the three-chambered social approach test used in this study (Grayton et al., 2013). Reported herein, we found that Nrxn1α HET mice had similar levels of sociability to WT mice, but did not show discrimination between a novel versus familiar mouse (i.e., Stranger 2 vs. Stranger 1). A limitation of this study was that we used only male adult mice as novel conspecifics, which could theoretically result in different inclinations toward social approach between male and female Nrxn HET mice. However, extensive validation using the same apparatus found no biasing effect of using male conspecifics; specifically there were “no confounding sexual or aggressive behaviors, because the strangers were contained within the wire cages” (Moy et al., 2004). Future studies may examine whether social impairments exist if using juvenile and/or same-sexed novel conspecifics.

The order in which we undertook our battery of tests could have theoretically influenced the results of some of the behaviors, particularly by using the more stressful tail suspension and forced swim tests before the social approach test. However, we think that the influence of this testing strategy would have been unlikely to confound our results. First, all genotypes had the same stress-related experiences; thus, the social approach test should have been equally affected. Second, all genotypes showed similar anxiety responses, meaning it is unlikely that baseline anxiety differences would influence subsequent testing. Third, there have been various approaches to the development of battery testing (Crawley & Paylor, 1997); Shoji et al. (2012), for example, subjected two lines of genetically altered mice to a comprehensive test battery that included the forced swim test 5 days before the social approach test. Given that the behavioral impairments we observed are relatively mild and specific, and that we provided rest periods (>1 week) between experiments, we argue that our findings are representative.

Differences exist across a range of other phenotypes in Nrxn1α HET mice within and between studies published thus far and the present work. Previously, Nrxn1α HET mice were found to be hyperactive in an open field and lacked novel object discrimination (Grayton et al., 2013; Laarakker et al., 2012), a finding that was not replicated in our study, although our study used an arena of slightly smaller dimensions. Nrxn1α KO mice have previously been shown to have impaired PPI and increased self-grooming (Etherton et al., 2009), phenotypes that were also not seen in the present Nrxn1α HET mice. Furthermore, despite examining each experiment for sex differences, we only observed a female-specific Nrxn1α HET impairment in the passive avoidance test. Previously, others have noted a male-specific hyperactivity phenotype in Nrxn1α HET mice (Laarakker et al., 2012), a profile that was not replicated in our study. The mechanisms underpinning sex differences caused by the α-neurexin I deletion are currently undefined, but further work is warranted in this area.

It is unclear why the results across these studies are different. In Grayton et al. (2013), the authors state that they backcrossed the Nrxn1α KO line eight times to derive a purer C57BL/6J background, which may have provided clearer behavioral results. The mice in the present study were a mix of C57BL/6NCrl (from the outcross at Leeds), 129 × 1/SvJ and an unspecified substrain of C57BL/6 before deposition at the Jackson Laboratory. It is possible that genetic background may influence the degree of impairment in the observed behaviors. However, given several independent laboratories have observed phenotypic behavioral alterations in Nrxn1α KO mice, it is likely that the gene itself is modulating the expression of normal behaviors. Hence, maintaining the neurexin deletion on a mixed genetic background is unlikely to be the sole explanation for the differences in behavioral alterations between the two studies. This idea has some basis given the variability of the clinical phenotypes linked to *NRXN1* deletions, which include autism, schizophrenia, mental retardation, and speech delay (Ching et al., 2010), with patient populations that have mixed genetic backgrounds compared with repeatedly backcrossed mouse strains. Furthermore, there are examples of *NRXN1* deletions that cause no observable clinical phenotype (Dabell et al., 2013), highlighting the variable penetrance of *NRXN1* deletions. To take social behaviors as an example, Nrxn1α deficient mice have shown a range of social impairments including poor maternal behaviors (Geppert et al., 1998), increased social aggression (Grayton et al., 2013) and a lack of social recognition (this study). Although further work is required to fully understand which social behaviors are altered by the loss of α-neurexin I, these studies demonstrate that this mouse model does replicate several symptoms of autism, thus, making it useful experimentally.

Previously we and others have examined Nrxn2α KO mice and found they expressed heightened anxiety-like behaviors and lacked social responses (Born et al., 2015; Dachtler et al., 2014). Within the current study, we examined Nrxn2α HET mice in the same behavioral tests, using identical protocols and apparatus, and, apart from reduced time in the center zone of the elevated plus maze, found that these mice do not have any anxiety-like phenotypes. They also had weaker social impairment phenotypes than their KO counterparts, although they did show significantly worse social approach between Stranger 1 and the empty cage (Figure 1c) and social preference between Stranger 2 and Stranger 1 (Figure 1d) in the three-chamber social test. However, the interpretations of this result are not clear given that only the discrimination ratio revealed significant differences, and social approach and social preference are more commonly presented as contact time between the two cages (Moy et al., 2004), which did not show any significant differences (Figure 1a–b).

Therefore, when not influenced by a strong anxiogenic phenotype, Nrxn2α HET mice show a subtle impairment in social tasks, potentially confirming Nrxn2 deletion as causing changes in social motivation that is not solely driven by their heightened anxiety (Dachtler et al., 2014). It is unclear why there is such a striking difference between Nrxn2α KO and Nrxn2α HET mice. In general, the dosage effect varies between genes, but in HET mice a functional copy usually makes the phenotype more benign and closer to WT. The molecular mechanism for the Nrxn2α KO and Nrxn2α HET mouse phenotypes remains to be explained but one suggestion is that it could be related to the expression of Munc18-1. Thus, in Nrxn2α KO mice (Dachtler et al., 2014), but not Nrxn2α HET mice (this study), there was lower expression of Munc18-1 within the hippocampus. As Munc18-1 HET mice have previously been shown to have increased anxiety phenotypes (Hager et al., 2014), it is possible that this is the mechanism responsible for the anxiogenic-like profile of Nrxn2α KO mice. We have previously shown that the hippocampal expression of genes related to synaptic function (*Stxbp1*, *Pvalb*, *Dlg4*, and *Grin2a*) was downregulated in Nrxn2α KO mice. In the current study, we found that the protein expression of Munc18-1, which was the only protein that significantly differed between WT and Nrxn2α homozygous KO mice (Dachtler et al., 2014), was not altered in Nrxnα HET mice. Elucidating which molecular pathways are impacted by the deletion of Nrxn genes will be an important future step in understanding how the genetic deletions result in aberant behaviors.

The deficits in sociability and social recognition in both Nrxn1α HET and Nrxn2α HET mice provide further evidence for the role of these genes in the genesis of behaviors relating to autism and schizophrenia. Given that the vast majority of α-neurexin deletions found thus far in autistic and schizophrenic patients are heterozygous (Reichelt et al., 2012), mice containing these deletions represent useful tools for further analysis of the biological pathways that contribute to the development of these conditions.

## Supplementary Material

10.1037/bne0000108.supp
