# Supplementary material for: Heterozygous Deletion of α-Neurexin I or α-Neurexin II Results in Behaviors Relevant to Autism and Schizophrenia
Source: Behav Neurosci. 2015 Dec;129(6):765–76. doi: 10.1037/bne0000108 (PMC4655861; doi:10.1037/bne0000108)
Supplement: Supplementary file 1 [file zeh005153042so1.doc]

**Supplemental Materials**

**Heterozygous Deletion of α-Neurexin I or α-Neurexin II Results in Behaviors Relevant to Autism and Schizophrenia**

**by J. Dachtler et al., 2015, *Behavioral Neuroscience***

**http://dx.doi.org/10.1037/bne0000108**

**Supplemental Figure Legends**

**
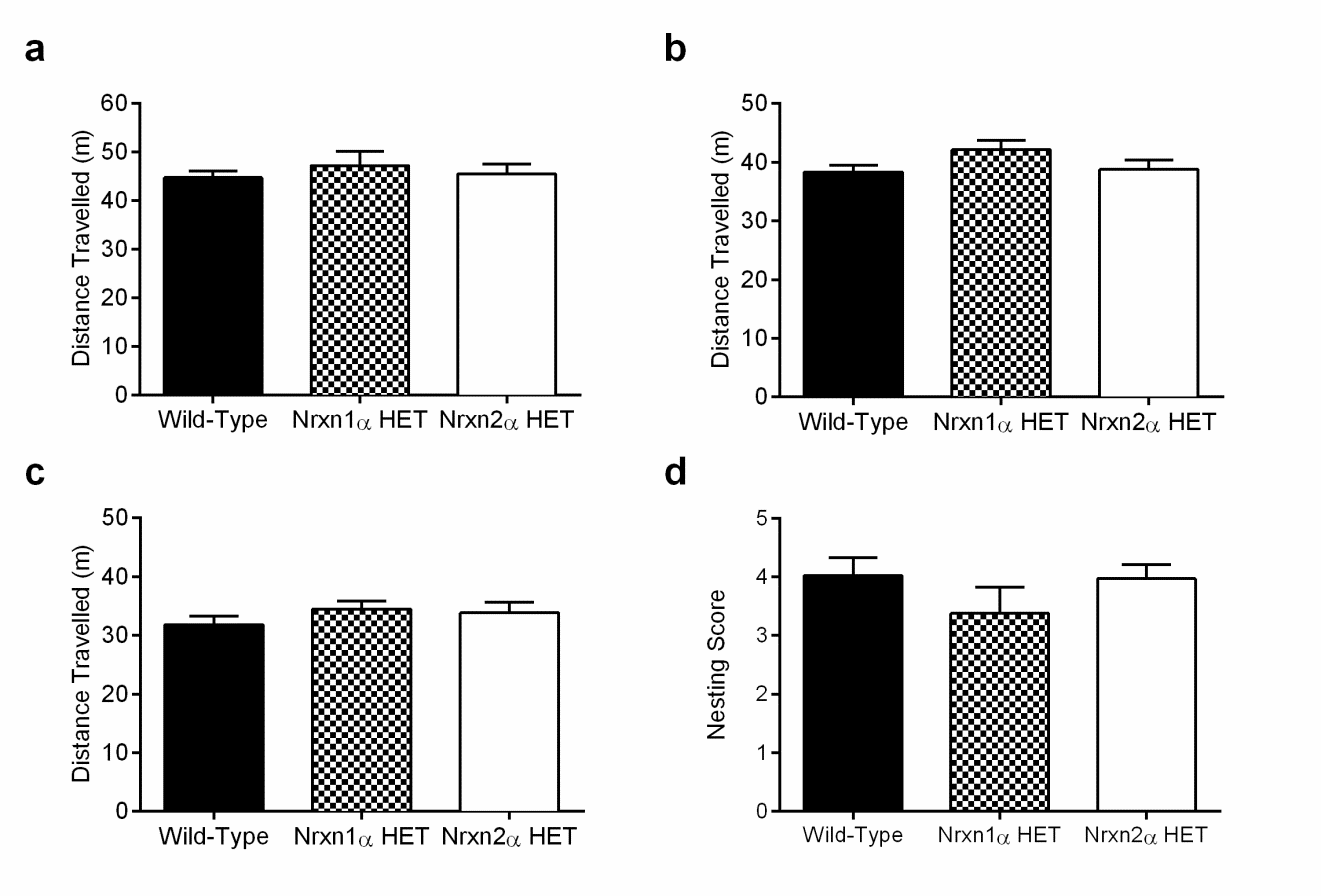
Figure S1**

**General ambulation was similar across all phases of the social interaction test.** Mice were tracked during the three phases of the social interaction test and the total distance travelled measured. No significant differences were observed during the habituation phase (a) (one-way ANOVA, F_(2, 62)_ <1), the ‘stranger 1 vs empty’ test (b) (one-way ANOVA, F_(2, 62)_ = 1.67, p > 0.05) or the ‘stranger 2 vs stranger 1’ test (c) (one-way ANOVA, F_(2, 62)_ <1). In a test of the ability of WT (*n* = 19), Nrxn1α HET (*n* = 12) and Nrxn2α HET (*n* = 16) to make nests within their home cage (d), no significant differences were observed.

**
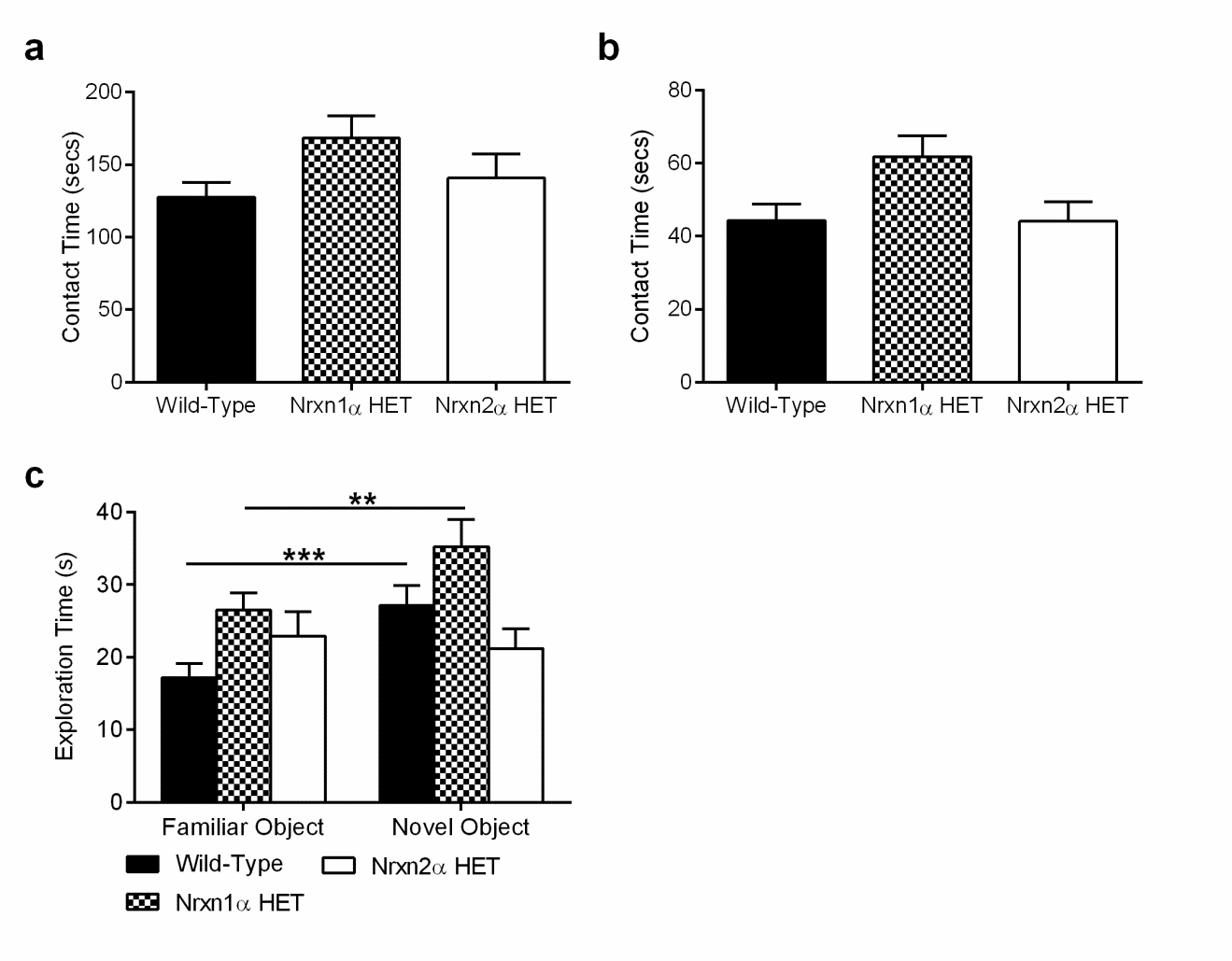
Figure S2**

**Contact time with the novel objects is not different between the genotypes.** During the acquisition phase, mice were presented with two objects and allowed to explore for 10 minutes (a). WTs, Nrxn1α HET and Nrxn2α HET mice made direct exploratory contact with the objects for similar amounts of time (one-way ANOVA, F_(2, 56)_ = 1.96, p > 0.05). 24 hours later, mice were presented with one of the previously explore objects and a novel object (b). Again, all genotypes explored the objects for a similar amount of time (one-way ANOVA, F_(2, 56)_ = 3.33, p = 0.043; pairwise comparison did not reveal any differences between the genotypes (all p > 0.05)). Time spent exploring the novel or familiar object revealed that WT and Nrxn1α HET showed statistically significant discrimination between the objects, while Nrxn2α HET mice did not (c) (RM two-way ANONA, genotype x object interaction F_(2, 56)_ = 7.52, p = 0.001, within genotype comparision: WT: p = 0.001; Nrxn1α HET: p = 0.006; Nrxn2α HET: p = 0.86).

**
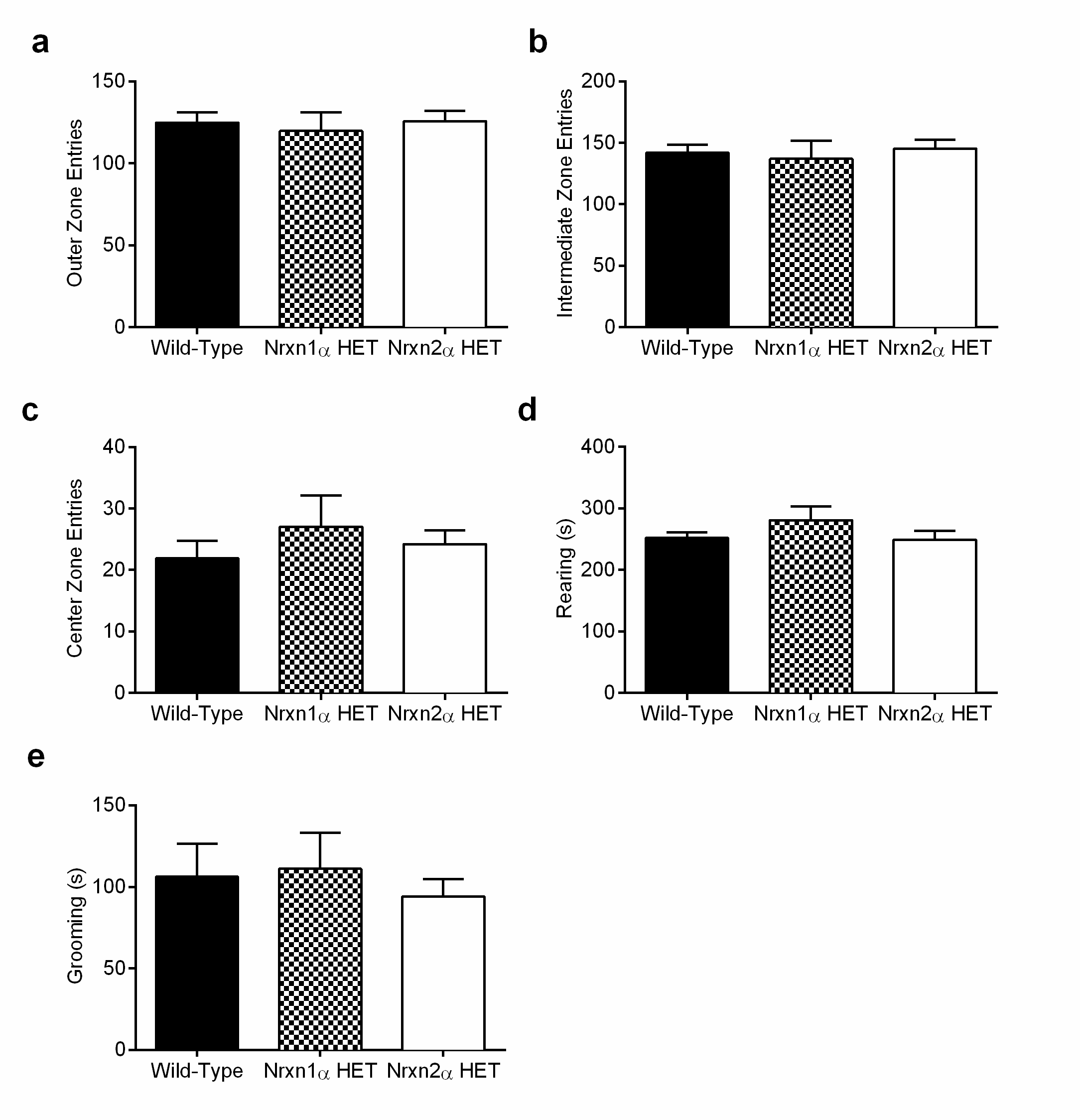
Figure S3**

**Exploratory behavior in a novel open field is similar between WT and Nrxn HET mice.** Mice were placed into a novel open field arena and their movements tracked over a 30 minute trial. The floor of the arena was divided into three zones. No significant differences were observed between the genotypes for the number of entries made into the outer zone (a), the intermediate zone (b) or the center zone (c) (one-way ANOVA, all F_(2, 62)_ <1). During the trial, the time the mouse spent rearing (d) and grooming (e) was measured. Nrxn1α HET and Nrxn2α HET mice spent similar times to WTs in both rearing (one-way ANOVA, F_(2, 62)_ = 1.14, p > 0.05) and grooming (one-way ANOVA, F_(2, 62)_ <1).

**
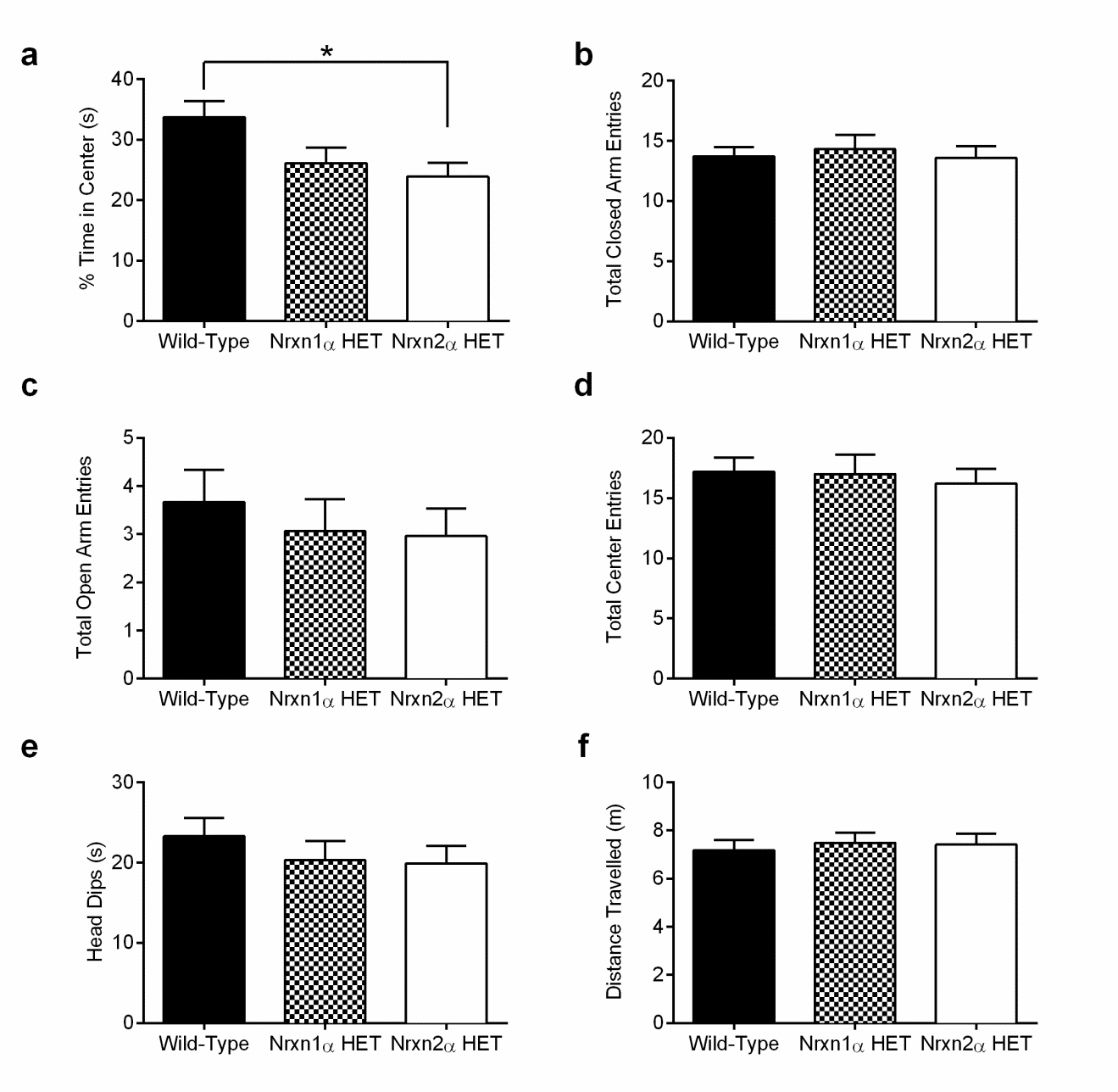
Figure S4**

**Exploratory behavior in the elevated plus maze is similar between the genotypes.** The tendency for mice to explore open arms verses closed arms was measured. Nrxn2α HET mice spent significantly less time within the center zone compared to WT mice (a) (one-way ANOVA, F_(2, 62)_ = 4.54, p = 0.014, pairwise comparisons for WT and Nrxn2α HET mice p = 0.013). No significant differences were found between the genotypes for the entries made into the closed arms (b) (one-way ANOVA, F_(2, 62)_ <1). Entries made into the open arms (c) and the center zone (d) were also similar between the genotypes (both one-way ANOVA, F_(2, 62)_ <1). All genotypes made a similar number of exploratory head dips (e) made over the center and open arms and travelled distance within the maze (f) (both one-way ANOVA, F_(2, 62)_ <1).

**
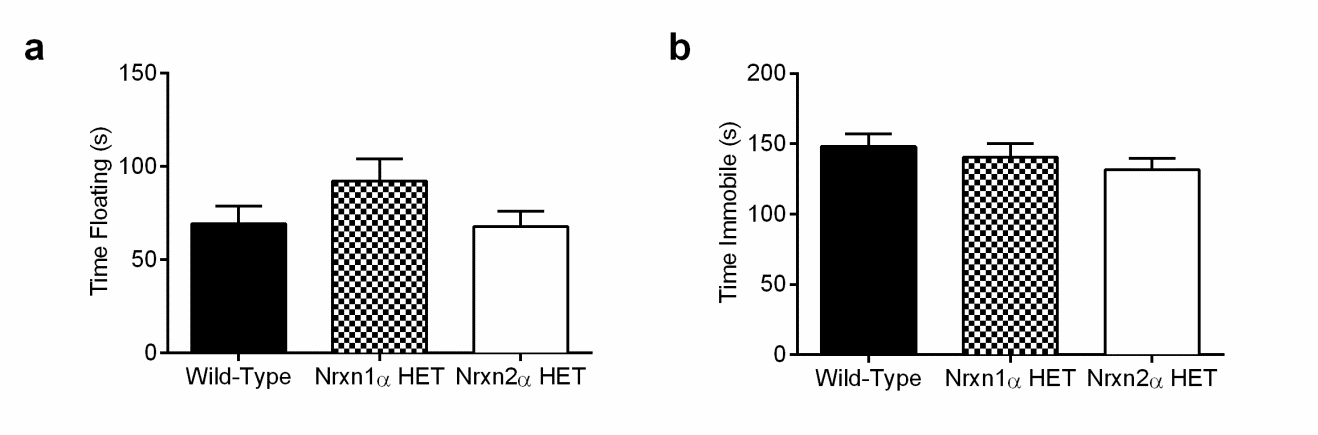
Figure S5**

**Nrxn HET mice are unaffected in measures of despair.** The propensity for Nrxn HET mice to float over a 6 minute trial was measured in the Porsolt forced swim test and was found to be similar between the genotypes (a) (one-way ANOVA, F_(2, 62)_ = 1.74, p > 0.05). Likewise, in the tail suspension test, Nrxn HET were immobile for a similar amount of time as WT mice (b) (one-way ANOVA, F_(2, 62)_ <1).
